# Editorial: “Building” health through physical activity in schools

**DOI:** 10.3389/fspor.2024.1359661

**Published:** 2024-01-18

**Authors:** Luís Branquinho, Pedro Forte, Ricardo Ferraz, José E. Teixeira, Andrew Sortwell

**Affiliations:** ^1^ Agrarian School of Elvas, Polytechnic Institute of Portalegre, Portalegre, Portugal; ^2^ Research Center in Sports Sciences, Health Sciences and Human Development, Covilhã, Portugal; ^3^ Centro de Investigação do ISCE (CI-ISCE), Ramada, Portugal; ^4^ Higher Institute of Educational Sciences of the Douro, Penafiel, Portugal; ^5^ Department of Sports Sciences and Physical Education, Polytechnic Institute of Bragança, Bragança, Portugal; ^6^ Sport Sciences Department, University of Beira Interior, Covilhã, Portugal; ^7^ Sport Department, Polytechnic Institute of Guarda, Guarda, Portugal; ^8^ School of Health Sciences and Physiotherapy, University of Notre Dame Australia, Sydney, NSW, Australia

**Keywords:** physical education, healthy behaviours, pedagogy, active methodologies, motivation

**Editorial on the Research Topic** “Building” health through physical activity in schools

Engaging in physical activity during childhood and adolescence is associated with improved physical and mental health (1, 2). Research has shown that physically active students exhibit better academic performance, a reduced likelihood of obesity, enhanced social skills, and higher levels of self-esteem. However, despite the many benefits of physical activity, many students do not engage in sufficient physical activity, and studies show that the levels of physical activity among children and adolescents have also decreased over time (3).

Most often, schools serve as more than just a centre for learning mandated curriculum (4). Within schools, the educators and the school communities also play a significant role in supporting the health and wellbeing of the learners, including supporting student participation in physical activity. However, in the school environment, barriers to physical activity include insufficient access to physical activity opportunities, limited time for recess, and lack of physical education, all of which can contribute to the problem of increased sedentary behaviours. In order to promote physical activity among students, it is important for schools to provide a variety of opportunities for physical activity and to make physical activity an integral part of the school day. Schools can take a proactive approach through curriculum, policies, and engagement with the school community to reverse the worsening trend of children and adolescents not meeting the recommended daily engagement of 60 min or more of moderate to vigorous physical activity.

Schools are considered one of the preferred intervention environments by government health authorities for increasing daily physical activity. The influence of school experiences and the environment on growth development and health status later in life is well documented (5). Evidence suggests that attitudes, beliefs, and behaviours learned during these early years—for example, those relating to physical activity—track into adulthood (6). Promoting engagement in physical activity and providing daily opportunities during these early formative years are important. Recognising this has led to an interest in using schools to promote and support physical activity behaviours in children and adolescents. Children spend a large proportion of their time at school, and thus, schools have the potential to be a robust domain of influence on children's health. In addition, a strong link exists between children's health status and their learning capacity (7). Therefore, creating positive and healthy school environments can improve health, wellbeing, and academic achievement, while also reducing inequities.

Consequently, helping schools fulfil their role of promoting physical activity should be a public health priority. The whole-school approach to promoting physical activity involves a comprehensive approach beyond conventional physical education and sports. A whole-school approach to enhance student health through physical activity involves prioritising regular and high-quality physical education classes, organising weekly school sports, encouraging active classrooms, and promoting active travel (walk/cycle-to-school programmes) to and from school. These actions should be supported by school policies and involve the entire school community (staff, students, parents). A whole-school approach is characterised by its commitment to promoting physical activity within the curriculum, school life, and the local community, aiming to cultivate physically literate and active individuals for life.

Not all students necessarily have the same opportunities to build their health through physical activity. Students with a disability have lower levels of physical activity compared with their typically developing peers (8). Hence, it is important to provide children with disabilities the opportunity to be more involved in suitable physical activities and sports. This importance is reinforced by the United Nations' Sustainable Development Goals (SDGs), particularly the goal of ensuring inclusive and quality education for all by 2030 (SDG 4) (9). The SDGs, established in 2015, serve as a global call to action to address various issues, including promoting equality and emphasising the need for integrative and equal-quality education to create opportunities for lifelong learning. In the context of children with disabilities, inclusive sports and physical activities play a crucial role in achieving this goal. Moreover, quality education is recognised as central to the overall success of the 2030 agenda, as education is interconnected with various other goals related to ensuring a healthy life and promoting the wellbeing of all people at all ages. In this context, physical activities contribute to the health and wellbeing of individuals, including children with disabilities.

Throughout this research topic dedicated to constructivist forms of health promotion through physical activity in schools, it is once again evident the crucial role that physical education plays in the construction of a healthy future for our children. Reflecting on the importance of regular exercise in the school environment leads us to realise that investing in the promotion of physical activity is not only a preventive measure for children, but also an investment in the integral and structured development of students. As we join forces to integrate the culture of physical activity into everyday school life, we contribute not only to healthier bodies, but also to more attentive, resilient, and capable minds to face the challenges of life. We hope that the information gathered in this research topic will serve as a catalyst for the transformation of schools in environments that truly promote better physical and mental wellbeing, thus building a solid foundation for a healthier and more balanced society.
